# Pre-pandemic mental health and coping strategy usage during the COVID-19 pandemic: a cross-sectional analysis of the Southern Cities Study

**DOI:** 10.1186/s12888-023-04987-3

**Published:** 2023-07-21

**Authors:** Gawon Cho, Daniel Hagen, Emily Goldmann

**Affiliations:** 1 Department of Social and Behavioral Sciences, School of Global Public Health, New York University, New York, USA; 2 Department of Epidemiology, School of Global Public Health, New York University, New York, NY USA; 3 Center for Drug Use and HIV|HCV Research, School of Global Public Health, New York University, New York, NY USA

**Keywords:** Mental health, Coping, COVID-19, Pandemic

## Abstract

**Background:**

Little is known about the usage of coping strategies recommended by the World Health Organization and the Centers for Disease Control and Prevention during the COVID-19 pandemic and whether coping strategy usage varies by pre-pandemic mental health. This study examined the prevalence of different coping strategies and associations of their usage with pre-pandemic mental health.

**Methods:**

Data were collected from adults residing in metropolitan areas of the U.S. South in May/June 2020 using random-digit-dialing and web-based surveys (*n* = 1,644). We estimated the prevalence of each coping strategy: (1) keeping up-to-date about COVID-19; (2) taking breaks from the news or social media; (3) taking care of physical health; (4) engaging in relaxing activities; (5) reaching out to and spending time with others; and (6) trying to find comfort in religious or spiritual beliefs. We examined the association between the use of each strategy and pre-pandemic mental health using modified Poisson regression, adjusting for covariates. We also analyzed the association between pre-pandemic mental health and the number of coping strategies employed using ordered logistic regression.

**Results:**

The most prevalent strategies were: “keeping up-to-date about COVID-19” (53%), “taking care of physical health” (52%), and “reaching out to and spending time with others” (52%). Good pre-pandemic mental health was associated with an increased prevalence of “reaching out to and spending time with others” (adjusted prevalence ratio, 1.43; 95% confidence interval, 1.07–1.91). The use of other coping strategies and the number of coping strategies used during the pandemic did not vary by pre-pandemic mental health.

**Conclusions:**

Our findings suggest that people who had good pre-pandemic mental health were more likely to connect with other people during the COVID-19 pandemic. Given the well-documented impact of social support on mental health in disaster contexts, efforts to promote safe social connections for those with pre-existing mental health concerns are needed.

**Supplementary Information:**

The online version contains supplementary material available at 10.1186/s12888-023-04987-3.

## Background

The COVID-19 pandemic created a series of unprecedented mental health challenges [1]. Subsequent to the onset of the pandemic, there substantial increases in the prevalence of symptoms of poor mental health were observed among adults residing in the United States and elsewhere [1–4]. A systematic review of data from U.S. nationally representative surveys conducted between March 2020 and July 2021 found a prevalence of moderate or severe depression or psychological distress of 39 percent among adults [2]. During the height of the pandemic, in late June 2020, symptoms of a depressive or anxiety disorder were present among 24 or 26 percent of the general population, respectively [3]. Eleven percent reported having considered suicide in the prior 30 days [3].

Public health authorities responded to these mental health challenges by providing the general public with a series of recommendations on coping with stress related to the pandemic, i.e., behaviors and thoughts used to manage stressful situations [5]. Strategies recommended by the World Health Organization (WHO) and the Centers for Disease Control and Prevention (CDC) include: engaging in relaxing activities; connecting with others; keeping up-to-date with COVID-19; taking care of one’s body, among others [6, 7]. However, little is known about the extent to which U.S. adults employed each of these coping strategies (i.e., a series of actions aimed at accomplishing a goal) during the pandemic. Previous research has examined the prevalence of standardized coping *styles* (i.e., general tendencies in coping with stress) in U.S. and non-U.S. samples [8–12]. Such research classified individuals dichotomously into those who use adaptive and maladaptive coping styles, which can help identify populations that are potentially at risk of psychiatric disorders, but may be limited in terms of identifying actionable areas for mental health promotion.

Moreover, little is known about differences in the use of coping strategies by pre-COVID-19 mental health status. Individuals with pre-existing psychiatric disorders have been found to experience a heavier burden of psychological distress during the early COVID-19 pandemic [13], which may, at least in part, be attributed to differences in coping. This idea is supported by a study of Dutch adults during the pandemic, which found a reduced likelihood of positive coping (e.g., “It is no problem to enjoy myself while being at home more often”; “Despite the virus I actively maintain (via phone or online) contacts with friends”) in individuals with a history of pre-existing psychiatric disorders compared to those without [14]. However, the study examined “positive coping” as a single, generalized style, leaving differences in coping strategies unexamined. In order to identify specific areas in need of support among individuals with a history of psychiatric disorders, empirical evidence is needed on how the use of coping *strategies* varied by pre-pandemic mental health status.

Hence, this research first sought to describe the prevalence of each coping strategy recommended by either the CDC or the WHO in a representative sample of adults residing in five Southern metropolitan areas of the U.S. Specifically, the following strategies were examined:

1. keeping up-to-date about COVID-19;
2. taking breaks from watching, reading, or listening to the news or social media;
3. taking care of physical health;
4. engaging in relaxing activities;
5. reaching out to or spending time with others; and
6. trying to find comfort in religious or spiritual beliefs.

Although the third strategy (i.e., trying to find comfort in religion) had not been explicitly recommended by public health authorities, it has been identified by prior research as an important method of coping in some subpopulations both prior to and during the pandemic [15, 16]. We also examined this strategy because the specific geographic region of interest in our study, the U.S. South, has a high proportion of adults who identify as being highly religious [17]. In addition, this research sought to analyze whether coping strategy usage varied by pre-COVID-19 mental health status. We analyzed the use of each strategy recommended by public health authorities as well as the total number of strategies used. Utilizing multiple coping strategies has been associated with an improved ability to adapt to stressful life situations by prior research [18]. Resulting findings may inform public health messaging during future infectious disease outbreaks, and help identify potential areas of support for vulnerable population subgroups. For example, adaptive coping strategies can alleviate symptoms of psychological distress and mental disorders [19, 20], including those evoked by sustained disasters such as the COVID-19 pandemic [21, 22].

## Methods

### Study design, participants, and data collection

This study used data collected in a cross-sectional survey of a probability-based sample of adults (18 years or older) residing in the following metropolitan statistical areas (MSAs) in the U.S. South: Atlanta-Sandy Springs-Alpharetta (Georgia), Austin-Round Rock-Georgetown (Texas), Dallas-Fort Worth-Arlington (Texas), Houston-The Woodlands-Sugar Land (Texas), and New Orleans-Metairie (Louisiana). The sample included participants recruited through both telephone and web-based sampling. The phone-based sampling method included random-digit-dial calls to residents within the metropolitan statistical areas of interest. The online respondents were randomly selected from the Dynata (formerly Survey Sampling International) panel of individuals residing in the metropolitan statistical areas of Atlanta, Austin, Dallas, Houston, and New Orleans. Respondents were asked about COVID-19-related experiences, mental health, and socio-demographic characteristics using either a web-based Qualtrics® survey or a random-digit-dial phone survey. Data collection took place between May 26 and June 6, 2020 (*n* = 1,644) [4]. Participants provided informed consent prior to beginning the survey. Details on recruitment, sample characteristics, and the survey questionnaires have been reported elsewhere [4].

### Measures

The outcomes of this study identified whether a respondent used a given coping strategy during the pandemic. Six dichotomous indicators (yes/no) were used to identify the use of each of the following strategies:

1. keeping up-to-date about COVID-19;
2. taking breaks from watching, reading, or listening to the news or social media;
3. taking care of physical health;
4. engaging in relaxing activities;
5. reaching out to or spending time with others; and
6. trying to find comfort in religious or spiritual beliefs.

During the survey, respondents were prompted, “The COVID-19 outbreak can be stressful to people. Since the outbreak began, what strategies have you used to cope with stress?” In response to this prompt, participants could select as many options as they wanted from a predefined list of individual coping methods detailed in Supplement 1. Based on the survey responses, we identified the users of each of the six coping strategies. The six coping strategy outcomes were characterized by reviewing pamphlets and websites on coping strategies provided by the WHO [6] and CDC [7], and identifying different dimensions of coping from the pamphlets and websites. Subsequently, the authors independently classified each of the survey response options in Supplement 1 into one of the dimensions. Differences in the characterization of dimensions and the classification of response options were resolved through consensus. Among the six finalized strategies, [1, 2], and [6] were mentioned verbatim in the response choices, whereas strategies [3, 4], and [5] were created by combining two or more individual coping methods listed in the answer choices (Supplement 1). Specifically, respondents were considered to have used “ [3] taking care of physical health” as a coping strategy during the pandemic if they reported at least one of the following: exercising; eating healthy; and getting enough sleep. Similarly, “ [4] engaging in relaxing activities” comprised activities to relax or to take one’s mind off things, meditating, and spending time outside in nature. Finally, “ [5] reaching out to or spending time with others” included: talking to friends and family about one’s feelings; connecting with others via text, phone, or online, spending time with others in the household; and talking to a counselor or health care provider about one’s feelings. In addition to these dichotomous outcomes, this study examined the total number of distinct coping strategies used during the pandemic (out of a possible 6 strategies). The number of coping strategies used was top-coded at five since only one person reported using all six strategies, resulting in the following categories: 0, 1, 2, 3, 4, and 5 + strategies.

The exposure of interest in this analysis was pre-pandemic mental health status, assessed by asking respondents: “How would you rate your overall mental/emotional health before the coronavirus/COVID-19 crisis?” Participants could respond using a Likert scale with five levels: poor, fair, good, very good, and excellent. In this study, participants were classified dichotomously into poor and fair vs. good, very good, and excellent, following prior research [23, 24]. The categories are henceforth referred to as “poor” and “good”.

Other covariates included gender, race/ethnicity, age, educational attainment, marital status, and self-rated general health. Gender was classified into men and women, and race/ethnicity was classified as follows: non-Hispanic White, non-Hispanic Black, Hispanic (any race), and non-Hispanic Asian. Age was categorized as follows: 18–24 years, 25–34 years, 35–44 years, 45–64 years, and 65 years or older. Educational attainment included: high school diploma (or equivalent) or less; Associate degree or some college; Bachelor’s degree; and post-graduate training or degree. Marital status included: married or cohabitating; single and never married; and divorced, separated, or widowed. Self-rated general health was classified dichotomously as good (good, very good, or excellent) vs. poor (poor or fair) [4]. We accounted for self-reported general health since it may affect an individual's access to some of the coping strategies, e.g., exercise or interacting with others [25].

### Statistical analysis

We first examined the absolute and relative distributions of each coping strategy outcome, the total number of coping strategies used during the pandemic, pre-pandemic mental health status, and covariates. Bivariate associations were examined by means of cross-tabulations and Rao-Scott tests. The results of bivariate analyses informed the identification of covariates to be included in the subsequent multivariate analyses (Supplement 2 and 3). Specifically, covariates were included if they were associated with both the exposure and at least one of the outcomes in bivariate analyses and/or considered to be potential confounders based on prior literature [13, 14, 18]. We then estimated the association between using a given coping strategy during the pandemic and pre-pandemic mental health without and with adjustments for covariates. Separate modified Poisson regression models were employed to analyze each of the six indicators. Prevalence ratios (PRs) and corresponding 95% confidence intervals (CIs) were reported. Model fit was assessed using deviance goodness-of-fit and the C-statistic (area under the Receiver Operating Curve (ROC) [26]. Of note, we chose to use modified Poisson regression with robust standard errors over logistic regression since odds ratios would overestimate prevalence ratios due to the high prevalence of coping strategies in the analytic sample [27–29]. We also examined the association between the total number of coping strategies used during the pandemic and pre-COVID-19 mental health status using ordered logistic regression with six ordinal categories, since our outcome is ordinal in nature (0, 1, 2, 3, 4, 5 + strategies). We examine this association without and with adjustments for the same series of covariates included in the models examining dichotomous indicators. Odds ratios (ORs) and 95% CIs are reported. We selected ordinal regression over Poisson regression since each coping strategy is not a discrete event and does not have a Poisson distribution.

In addition to the full sample, we also conducted gender-stratified analyses. Prior research has demonstrated substantial differences in the prevalence of psychiatric disorders and the use of coping strategies between men and women [30–32]. All analyses were conducted using complex survey procedures including survey weights to approximate the target population in each MSA with respect to age, gender, race/ethnicity, and educational attainment. The processes involved in weight development are reported elsewhere [4]. All statistical analyses were conducted using Stata/SE 17.0, and statistical significance was evaluated at *p* < 0.05 throughout.

## Results

Table 1 shows the characteristics of the study sample (*n* = 1,644). The sample was equally composed of men and women. Forty-four percent of respondents were non-Hispanic White, 27% were non-Hispanic Black, 27% were Hispanic (any race), and 7% were non-Hispanic Asian. Thirty percent of participants had attended some college or obtained an Associate’s degree, 24% had obtained a Bachelor’s degree, and 14% had undergone post-graduate training or obtained a postgraduate degree. Fifty-three percent were married or cohabitating, 33% were single and had never been married, and 14% were divorced or separated. The vast majority (85%) of respondents rated their self-reported general health as either good, very good, or excellent. Eleven percent reported having poor pre-pandemic mental health status, while 89% reported having good mental health prior to the pandemic. In terms of coping strategies in the wake of the COVID-19 outbreak, 53% reported keeping up-to-date about COVID-19; 24% said they took breaks from the news or social media; 52% said they took care of their physical health; 39% reported engaging in relaxing activities; 52% reported having reached out to and/or spent time with others; and 16% reported having tried to find comfort in religious or spiritual beliefs. Overall, the median number of coping strategies used during the pandemic was two (interquartile range: 1–3).

**Table 1:** Characteristics of respondents to the Southern Cities Study, 26 May to 6 June, 2020 (*n* = 1,644)

Characteristics	N (%)^a^ / Med [IQR]
Gender	
Men	790 (49.9)
Women	850 (50.1)
Age category (years)	
18 – 24	287 (12.1)
25 – 34	306 (20.6)
35 – 44	259 (18.6)
45 – 64	453 (32.5)
65 – 89	339 (16.3)
Race/ethnicity	
Non-Hispanic White	554 (44.3)
Non-Hispanic Black	476 (26.6)
Hispanic, any race	361 (26.6)
Non-Hispanic Asian	219 (7.4)
Educational attainment	
High school diploma (or equivalent) or less	279 (31.9)
Associate degree or some college	462 (29.6)
Bachelor’s degree	528 (24.3)
Post-graduate training or degree	375 (14.2)
Marital status	
Married or cohabitating	874 (52.5)
Single, never married	527 (33.2)
Divorced or separated	231 (14.3)
Self-rated general health	
Good, very good, or excellent	1,435 (85.3)
Poor or fair	181 (14.7)
Self-rated pre-pandemic mental health	
Good	1,454 (88.9)
Poor	155 (11.1)
Coping strategies since COVID-19 outbreak *(multiple responses possible)*	
Keeping up-to-date about COVID-19	840 (52.9)
Taking breaks from watching, reading, or listening to news or social media	342 (23.5)
Taking care of physical health^b^	829 (51.8)
Engaging in relaxing activities^c^	616 (38.7)
Reaching out to and/or spent time with others^d^	805 (52.1)
Trying to find comfort in religious or spiritual beliefs	263 (16.0)
Total number of coping strategies endorsed	
0	65 (6.0)
1	322 (21.6)
2	377 (24.0)
3	481 (30.0)
4	250 (16.6)
5 or 6	35 (1.8)

*Acronyms*: *Med* Median, *IQR* Interquartile range

^a^Absolute and weighted relative frequencies

^b^Exercised, ate healthy, and/or tried to get enough sleep

^c^Did activities to relax / take mind off things, meditated, and/or spent time outside in nature

^d^Talked to friends and family about feelings, connected with others via text/phone/online, spent time with others in household, and/or talked to a counselor or health care provider about feelings

Table 2 shows the estimates of unadjusted and adjusted associations between using a given coping strategy and having good pre-COVID-19 mental health from separate modified Poisson regression models. In unadjusted models, good pre-pandemic mental health was associated with significantly greater prevalence of “keeping up-to-date about COVID-19” (PR = 1.33, 95%CI = 1.01–1.75) and “reaching out to or spending time with others” (PR = 1.45, 95%CI = 1.09–1.92). After adjustments for all covariates, pre-pandemic mental health was statistically significantly associated with “reaching out to or spending time with others” only. Specifically, individuals with good pre-pandemic mental health had a 43% higher prevalence of using this strategy during the pandemic than individuals with poor pre-pandemic mental health in the full analytic sample (PR = 1.43, 95% CI: 7–91%). However, it should be noted that, in the adjusted gender-stratified analyses, the difference in the prevalence of “reaching out to or spending time with others” by pre-pandemic mental health remained significant in women only, after adjusting for covariates (PR = 1.61, 95%CI = 1.05–2.46). The association was not statistically significant among men (PR = 1.25, 95%CI = 0.86–1.83). The prevalence of the other five coping strategies did not show significant differences by pre-pandemic mental health status in men or women. Deviance goodness-of-fit tests provided no evidence of model misspecification (with *p* = 1.000).

**Table 2:** Associations of coping strategy use and good pre-pandemic mental health status, Southern Cities Study, 26 May to 6 June, 2020

Sample	Coping strategies	Unadjusted PR [95% CI]	Adjusted PR^a^ [95% CI]
Full sample			
	Keeping up-to-date about COVID-19	1.33 [1.01;1.75]*	1.26 [0.95;1.66]
	Taking breaks from watching, reading, or listening to news or social media	1.29 [0.77;2.14]	1.13 [0.68;1.90]
	Taking care of physical health	1.28 [0.98;1.68]	1.12 [0.84;1.49]
	Engaging in relaxing activities	0.91 [0.69;1.21]	0.89 [0.67;1.17]
	Reaching out to / spent time with others	1.45 [1.09;1.92]*	1.43 [1.07;1.91]*
	Trying to find comfort in religious or spiritual beliefs	1.33 [0.71;2.50]	1.19 [0.64;2.20]
Men			
	Keeping up-to-date about COVID-19	1.14 [0.80;1.64]	1.10 [0.78;1.54]
	Taking breaks from watching, reading, or listening to news or social media	1.48 [0.64;3.40]	1.41 [0.60;3.34]
	Taking care of physical health	1.03 [0.72;1.47]	0.93 [0.66;1.31]
	Engaging in relaxing activities	1.06 [0.70;1.63]	1.11 [0.71;1.73]
	Reaching out to / spent time with others	1.23 [0.83;1.83]	1.25 [0.86;1.83]
	Trying to find comfort in religious or spiritual beliefs	2.14 [0.71;6.44]	2.04 [0.74;5.64]
Women			
	Keeping up-to-date about COVID-19	1.54 [1.02;2.33]*	1.44 [0.95;2.18]
	Taking breaks from watching, reading, or listening to news or social media	1.16 [0.61;2.22]	0.95 [0.51;1.77]
	Taking care of physical health	1.59 [1.06;2.40]*	1.34 [0.87;2.07]
	Engaging in relaxing activities	0.82 [0.57;1.17]	0.76 [0.53;1.10]
	Reaching out to / spent time with others	1.70 [1.14;2.52]**	1.61 [1.05;2.46]*
	Trying to find comfort in religious or spiritual beliefs	1.15 [0.55;2.41]	0.98 [0.48;1.99]

Reference category = poor pre-pandemic mental health

*Acronyms*: *PR* Prevalence ratio, *CI* Confidence interval

Significance levels: * *p* < 0.05; ** *p* < 0.01

^a^Adjusted for: gender (full sample only), age, race/ethnicity, educational attainment, marital status, self-rated general health

Finally, Table 3 shows the estimates of unadjusted and adjusted associations between pre-pandemic mental health and the total number of coping strategies used during the pandemic. Prior to adjusting for covariates, having good pre-pandemic mental health was statistically significantly associated with increases in the number of coping strategies used in the full sample (OR = 2.06, 95%CI: 1.22–3.48) and among women (OR = 2.48, 95%CI = 1.32–4.68), but not among men (OR = 1.72, 95%CI: 0.66–4.47). After adjustments for covariates, the estimates were no longer significant in the full sample and the women subsample. Approximate likelihood-ratio test of proportionality showed that the proportionality assumption was not violated.

**Table 3:** Associations of the total number of coping strategies used and good pre-pandemic mental health status, Southern Cities Study, 26 May to 6 June, 2020

	**Unadjusted OR [95% CI]**	**Adjusted OR **^**a**^** [95% CI]**
Full sample	2.06 [1.22;3.48]**	1.68 [0.97;2.90]
Men	1.72 [0.66;4.47]	1.65 [0.61;4.42]
Women	2.48 [1.32;4.68]**	1.88 [0.96;3.69]

Reference category = poor pre-pandemic mental health

*Acronyms*: *OR* Odds ratio, *CI* Confidence interval

Significance levels: ** *p* < 0.01

^a^Adjusted for: gender (full sample only), age, race/ethnicity, educational attainment, marital status, self-rated general health

## Discussion

The use of effective coping strategies is especially important during sustained pandemics when individuals experience increased burden of psychological distress and of symptoms of mental disorders. While there is some prior research on the use of dichotomously-classified positive vs. negative coping styles, there is little evidence on the use of distinct coping strategies. Therefore, this study examined the use of coping strategies recommended by the WHO and CDC during the COVID-19 pandemic in a representative sample of adults residing in the U.S. South. The three most prevalent coping strategies used by more than half of the sample were: “keeping up-to-date about COVID-19”, “taking care of physical health,” and “reaching out to or spending time with others.” These strategies have problem-focused elements [33] that include active responses aimed at mitigating risks (i.e., COVID-19 infection) or inconveniences (i.e., quarantine) commonly experienced during the pandemic. Specifically, “keeping up-to-date about COVID-19” is aimed at reducing the risks of COVID-19 infection, while “taking care of physical health” is aimed at improving bodily health, which is also important in virus prevention. Furthermore, “reaching out to or spending time with others” aims to counter the negative impact of quarantine measures on social connectedness and psychological well-being. The following strategies were relatively less common: “taking breaks from watching, reading, or listening to the news or social media”; “trying to find comfort in religious or spiritual beliefs”; and “engaging in relaxing activities.” These strategies have emotion-focused elements, which include attempts to cope with stress by reducing negative psychological responses to a given stressor [33]. These results suggest that both problem- and emotion-focused strategies were commonly used to cope with stress during the early COVID-19 pandemic period.

Furthermore, we examined differences in coping strategy use by pre-pandemic mental health status, another area left unexamined by prior research. Our findings show that adults with poor pre-pandemic mental health were less likely to “reach out to or spend time with others” to manage their stress than their counterparts with good pre-pandemic mental health, net of sociodemographic characteristics and self-reported general health. We did not find any differences in the use of other strategies and the total number of strategies used after adjusting for covariates. Previously, having a pre-pandemic diagnosis of a mental disorder had been found to be associated with an increased burden of psychological distress during the pandemic [34]. We add to this line of research by showing that adults with poor pre-pandemic mental health were less likely to use coping strategies focused on social interaction. Since social disconnection can negatively affect mental health [35], decreased use of social interaction-based coping strategies may lead to increased psychological distress in adults with a pre-pandemic history of mental illness. Even in non-pandemic contexts, individuals with depression, anxiety, and other psychiatric disorders exhibit increased social inhibition and withdrawal [36, 37], and quarantine measures may have widened this disparity during the pandemic. Thus, during pandemics, interventions to promote safe forms of social interaction in adults with existing psychiatric disorders (e.g., virtual group activities) may be necessary. During the pandemic, grassroots initiatives (e.g., Survivor Corps [38], Body Politic [39]) focused on social and emotional support using online platforms. In contrast, state-funded programs tend to focus on providing professional counseling and resources rather than fostering social interactions, e.g., the Substance Abuse and Mental Health Service Administration Helpline [40] or the 988 Suicide and Crisis Lifeline [41]. The findings of this study on the importance of social interactions during COVID-19 suggest that state-funded coping programs should consider broadening their scope to address potentially unmet needs.

Finally, our gender-stratified analyses showed that poor pre-pandemic mental health was associated with a lower prevalence of social interaction-based coping strategy use only among women. During the COVID-19 pandemic, larger increases in the prevalence of psychological distress were observed in women with pre-existing psychiatric disorders than in their male counterparts [42, 43]. Our findings suggest that this gender difference may be attributed, at least in part, to the differences in coping strategy use by pre-pandemic mental health status being limited to women only. Future research may identify obstacles to using social interactions to cope with distress that may be specific to women with poor pre-pandemic mental health, potentially associated with changes in the division of domestic labor during the COVID-19 pandemic. Prior research has found increases in gender inequality in domestic labor during the pandemic [44].

This study is subject to several limitations. First, coping strategies were self-reported and may be subject to reporting bias, e.g., social desirability. Second, pre-COVID-19 mental health was assessed retrospectively after the pandemic started by means of a single, non-validated questionnaire item. Given the evidence of population-wide increases in the burden of psychiatric disorders during the pandemic [1, 2], retrospective mental health assessments may have led to a non-differential over- or under-reporting of poor pre-pandemic mental health. Hence, the measures of association may have been biased towards the null. Third, the study sample may not be entirely representative of adults residing in the South given that the majority of participants completed the survey online. Finally, this study focuses on coping strategies recommended by public health authorities. Future research may study other strategies that may be helpful in coping in prolonged disaster contexts that were not specifically suggested by the WHO or the CDC. On the other hand, this study has several strengths, including the use of a probability-based sample, examination of gender differences, and the inclusion of a variety of diverse coping strategies, which addresses a critical gap in research on coping during pandemics.

In sum, this study extends prior research that focused on the use of adaptive and maladaptive coping *styles* during the pandemic by examining the use of coping *strategies* recommended by public health authorities. Our findings suggest that a higher proportion of adults residing in the U.S. South used strategies to directly modify pandemic-related stressors (i.e., risk of infection and social disconnectedness). Moreover, we show that poor pre-pandemic mental health was associated with a lower prevalence of social interaction-based coping strategy use among women, but not among men. Future research may explore social interaction-based public health interventions for mental health promotion in disaster contexts, specifically in populations with existing psychiatric disorders and among women.

## Supplementary Information

**Additional file 1: Supplement 1.** Pre-defined list of coping methods. **Supplement 2.** Bivariate associations with coping strategies among respondents to the Southern Cities Study, 26 May to 6 June, 2020. **Supplement 3.** Bivariate associations with pre-pandemic mental health among respondents to the Southern Cities Study, 26 May to 6 June, 2020 †.

## Data Availability

The datasets generated and/or analyzed during the current study are not publicly available due to the fact that participants’ privacy could be compromised but are available from the corresponding author on reasonable request.
