# Supplementary material for: Pre-pandemic mental health and coping strategy usage during the COVID-19 pandemic: a cross-sectional analysis of the Southern Cities Study
Source: BMC Psychiatry. 2023 Jul 21;23:530. doi: 10.1186/s12888-023-04987-3 (PMC10362574; doi:10.1186/s12888-023-04987-3)
Supplement: Supplementary file 1 — Additional file 1: Supplement 1. Pre-defined list of coping methods. Supplement 2. Bivariate associations with coping strategies among respondents to the Southern Cities Study, 26 May to 6 June, 2020. Supplement 3. Bivariate associations with pre-pandemic mental health among respondents to the Southern Cities Study, 26 May to 6 June, 2020 †. [file 12888_2023_4987_MOESM1_ESM.docx]

**Supplement 1.** Pre-defined list of coping methods

1. Kept up-to-date about COVID-19

2. Did activities that help me relax/take my mind off things

3. Meditated

4. Tried to find comfort in my religion and spiritual beliefs

5. Talked to my friends and family about how I am feeling

6. Talked to a counselor or health care provider about how I am feeling

7. Exercised

8. Connected with other via text, phone or online

9. Ate healthy

10. Took breaks from watching, reading or listening to news or social media

11. Volunteered in my community

12. Tried to get enough sleep

13. Spent time with others in my household

14. Spent time outside in nature

15. Others (write in)

16. I have not used any strategies

**Supplement 2.** Bivariate associations with coping strategies among respondents to the Southern Cities Study, 26 May to 6 June, 2020**^†^**

| **Covariates** |  |  |  | **%** |  |  |
| --- | --- | --- | --- | --- | --- | --- |
|  | **Kept up-to-date** | **Physical health^1^** | **Time with others^2^** | **Relaxing activities^3^** | **Breaks from news** | **Religious/spiritual beliefs** |
|  |  |  |  |  |  |  |
| Full sample | 52.9 | 51.8 | 52.1 | 38.7 | 23.5 | 16.0 |
| Gender |  |  |  |  |  | * |
| Male | 54.7 | 50.6 | 51.1 | 38.3 | 23.2 | 12.9 |
| Female | 50.8 | 53.2 | 52.8 | 39.1 | 23.8 | 19.2 |
| Age category (years) | *** | *** | *** |  | *** | * |
| 18 – 24 | 50.8 | 43.8 | 45.6 | 38.5 | 10.6 | 11.6 |
| 25 – 34 | 47.5 | 43.9 | 39.4 | 37.2 | 14.5 | 10.0 |
| 35 – 44 | 48.1 | 46.4 | 47.7 | 48.5 | 18.6 | 14.1 |
| 45 – 64 | 50.7 | 56.0 | 56.8 | 36.3 | 34.6 | 20.1 |
| 65 – 89 | 70.6 | 65.3 | 67.7 | 34.4 | 27.3 | 20.3 |
| Race/ethnicity | ** | *** | ** |  | ** | ** |
| Non-Hispanic White | 57.4 | 60.1 | 57.1 | 39.1 | 28.1 | 18.0 |
| Non-Hispanic Black | 50.4 | 40.3 | 43.3 | 34.4 | 14.9 | 21.4 |
| Hispanic, any race | 44.0 | 46.3 | 50.8 | 43.3 | 23.8 | 10.0 |
| Non-Hispanic Asian | 61.5 | 50.2 | 43.5 | 31.4 | 18.9 | 14.4 |
| Educational attainment | * | *** | ** | * |  |  |
| High school diploma  (or equivalent) or less | 45.8 | 39.9 | 45.3 | 32.1 | 17.7 | 15.5 |
| Associate degree or some college | 52.9 | 52.0 | 49.9 | 39.0 | 25.4 | 16.8 |
| Bachelor’s degree | 60.6 | 57.8 | 57.4 | 42.2 | 27.8 | 19.3 |
| Post-graduate training or degree | 54.9 | 67.3 | 62.0 | 46.6 | 24.8 | 9.9 |
| Marital status | ** | ** | *** |  |  | ** |
| Married or cohabitating | 53.2 | 57.7 | 57.7 | 42.1 | 24.5 | 19.2 |
| Single, never married | 46.9 | 42.6 | 41.8 | 34.8 | 21.6 | 10.3 |
| Divorced or separated | 65.2 | 51.5 | 54.8 | 35.3 | 24.3 | 17.4 |
| Self-rated general health |  | * |  |  |  |  |
| Good, very good, or excellent | 53.0 | 53.9 | 51.5 | 39.9 | 23.9 | 16.1 |
| Poor or fair | 52.3 | 40.0 | 55.7 | 31.3 | 21.3 | 15.3 |
| Self-rated pre-pandemic mental health | * | * | ** |  |  |  |
| Good, very good, or excellent | 54.3 | 53.2 | 54.1 | 38.4 | 24.0 | 16.5 |
| Poor or fair | 40.8 | 41.5 | 37.3 | 41.9 | 18.7 | 12.4 |

† Results of weighted chi-square tests are indicated in covariate name rows

1. Exercised, ate healthy, and/or tried to get enough sleep
2. Talked to friends and family about feelings, connected with others via text/phone/online, spent time with others in household, and/or talked to a counselor or health care

provider about feelings

1. Did activities to relax / take mind off things, meditated, and/or spent time outside in nature

Significance levels: * *p*<0.05; ** *p*<0.01; *** *p*<0.001

**Supplement 3.** Bivariate associations with pre-pandemic mental health among

respondents to the Southern Cities Study, 26 May to 6 June, 2020 †

| **Covariates** | **Pre-pandemic mental health** | |
| --- | --- | --- |
|  | **Poor/fair** | **Excellent/very good/good** |
|  | **%^†^** | **%^†^** |
| Total | 11.1 | 88.9 |
| Gender |  |  |
| Male | 9.3 | 90.7 |
| Female | 12.8 | 87.2 |
| Age category (years)** |  |  |
| 18 – 24 | 10.3 | 89.7 |
| 25 – 34 | 12.3 | 87.7 |
| 35 – 44 | 17.1 | 82.9 |
| 45 – 64 | 11.4 | 88.7 |
| 65 – 89 | 3.0 | 97.0 |
| Race/ethnicity |  |  |
| Non-Hispanic White | 10.1 | 89.9 |
| Non-Hispanic Black | 12.5 | 87.5 |
| Hispanic, any race | 12.6 | 87.4 |
| Non-Hispanic Asian | 9.6 | 90.4 |
| Educational attainment |  |  |
| High school diploma (or equivalent)  or less | 11.7 | 88.3 |
| Associate degree or some college | 13.4 | 86.6 |
| Bachelor’s degree | 8.6 | 91.4 |
| Post-graduate training or degree | 9.2 | 90.8 |
| Marital status |  |  |
| Married or cohabitating | 9.4 | 90.7 |
| Single, never married | 13.8 | 86.2 |
| Divorced or separated | 11.6 | 88.4 |
| Self-rated general health*** |  |  |
| Good, very good, or excellent | 7.2 | 92.8 |
| Poor or fair | 33.6 | 66.4 |

† The table displays weighted relative frequencies. Results of weighted chi-square

tests are indicated next to each covariate.

Significance levels: * *p*<0.05; ** *p*<0.01; *** *p*<0.001
